# Characteristics of participants and nonparticipants in a population based diagnostic survey of mental and substance use disorders. A follow-up study of the Trøndelag Health Study (HUNT)

**DOI:** 10.1007/s00127-025-03030-y

**Published:** 2026-01-13

**Authors:** Ann Kristin Skrindo Knudsen, Jens Christoffer Skogen, Børge Sivertsen, Kristin Gustavson, Kim Stene-Larsen, Steinar Krokstad, Alize J. Ferrari, Ronald C. Kessler, Anne Reneflot

**Affiliations:** 1 Department of Disease Burden, Norwegian Institute of Public Health, Bergen, Norway; 2 Department of Health Promotion, Norwegian Institute of Public Health, Bergen, Norway; 3 Center for Alcohol & Drug Research, Stavanger University Hospital, Stavanger, Norway; 4 Centre for Evaluation of Public Health Measures, Norwegian Institute of Public Health, Oslo, Norway; 5 Department of Research & Innovation, Helse Fonna, Norway; 6 Department of Psychology, University of Oslo, Oslo, Norway; 7 Department of Childhood and Families, Norwegian Institute of Public Health, Oslo, Norway; 8 Department of Mental Health, Norwegian Institute of Public Health, Oslo, Norway; 9 Department of Public Health and Nursing, Norwegian University of Science and Technology, Trondheim, Norway; 10 School of Public Health, The University of Queensland, Brisbane, QLD Australia; 11 Queensland Centre for Mental Health Research, Brisbane, QLD Australia; 12 Institute for Health Metrics and Evaluation, University of Washington, Seattle, USA; 13 Department of Health Care Policy, Harvard Medical School, Boston, USA

**Keywords:** Selection bias, Mental health, Population-based surveys, Registry data, Nonparticipation

## Abstract

**Purpose:**

Participation rates in population-based health surveys have been declining for decades, with nonresponse bias being a serious threat to the validity and generalizability of results. The aim of the study was to examine differences between participants and nonparticipants invited to a psychiatric diagnostic interview survey in terms of sociodemographic and health characteristics, and use of health services for mental health problems, including diagnoses.

**Methods:**

The study sample for the follow-up psychiatric diagnostic interview was recruited among participants in the fourth survey of the Trøndelag Health Study (HUNT4) in Norway. Information about sociodemographic and health characteristics was gathered from the main survey, while data on health service use was collected by linking records from primary and specialist patient registries with information about participation status.

**Results:**

Male sex, younger age, being unmarried, having lower educational attainment, and having lower income were associated with higher odds for nonparticipation. Contact with the primary or specialist health services for mental health problems and hospitalization for a mental disorder more than a year before or after invitation date, were associated with lower odds for nonparticipation, especially for diagnoses indicating affective disorders, anxiety disorders, personality disorders, hyperkinetic disorders or milder mental complaints.

**Conclusion:**

Contrary to most prior studies examining nonresponse bias, the results indicate that people who had been in contact with the health services for mental health problems were more inclined to participate in a survey focused on mental disorders. The direction of nonresponse bias should be carefully considered and adjusted for in population-based studies.

## Introduction

Mental and substance use disorders are estimated to constitute a substantial disease burden [1], but knowledge about the prevalence and distribution of these disorders is limited by the lack of high quality epidemiological data [2]. Robust population-based prevalence estimates require assessments according to diagnostic criteria, which makes data-collection expensive and time-consuming. Hence, population-based studies assessing mental and substance use disorders are scarce. It is therefore important to ensure that the results from the few existing studies can be generalized to the target population.

Participation rates in population-based health surveys have been declining for decades [3–6]. For instance, the response rate in the Trøndelag Health Study (HUNT) has fallen from 89.4% in 1984-86 (HUNT1), to 54.0% in 2017–2019 (HUNT4) [7]. A recent systematic review of excluded groups and non-response in mental health surveys highlighted the systematic underrepresentation of young people and men as well as vulnerable populations, such as homeless individuals, and those in healthcare or correctional facilities [8]. A high nonparticipation rate is a serious threat to the representativeness, validity and generalizability of the collected data, as nonparticipation rarely is random. It is therefore essential to capture information related to characteristics that differ between participants and nonparticipants and consider their impact on data analyses and interpretation.

Obtaining high-quality information about nonparticipants is challenging, as this requires access to data on everyone invited to the survey, regardless of participation status. Some studies, such as the WHO World Mental Health Surveys, conduct nonresponse studies, where probability subsamples of initial non-responders are provided with incentives to complete short questionnaires to assess sociodemographic and mental health related characteristics [9]. However, such follow-up studies are plagued by low response rates, and have given inconsistent findings across surveys when comparing prevalence of mental distress between responders and non-responders [10, 11]. Another option is to study predictors of attrition between waves in longitudinal studies, with findings indicating poorer lifestyle (higher rates of smoking, risky alcohol consumption, and higher drug use), and poorer general, somatic and mental health among initial responders lost to follow-up [12]. Another possibility is to cross-reference participants’ information with aggregated data from official statistics or registries [13]. For example, a study from Finland indicated lower prevalence of heavy drinking among survey participants than the general population [14]. Also, a Danish study found slightly higher prevalence of previous diagnoses of organic mental or substance use disorders but similar prevalence of depressive disorders or anxiety disorders between the eligible population and survey respondents, particularly when survey weights were added [15].

Health surveys that draw their samples from population registries or censuses are able to obtain reliable demographic data on invited individuals, showing higher nonparticipation rates among men and younger individuals [13]. Studies using registry data linked on individual level with participation status have found that nonparticipation is often associated with lower socioeconomic position (lower income, lower educational attainment, poorer employment status, and being unmarried) [13], and measures indicating poorer health, such as higher rates of hospitalization, greater benefit recipiency, and higher mortality [13], including receiving permanent disability benefits due to mental and alcohol use disorders [16]. A recent Danish study linking persons invited to a large general population health study with registry data from psychiatric hospitals, found that nonparticipation was higher among those with psychiatric diagnoses, psychiatric hospital utilization, and psychotropic drug use [17]. This may indicate that nonparticipants are more prone to having mental health problems than participants, resulting in underestimation of mental health problems in surveys that do not adjust for these biases.

Norway has a long tradition of conducting large population-based health surveys. A key advantage of the Norwegian setting is the availability of high-quality data from several administrative and health registries, that can be linked to survey samples through individual national identification numbers. This provides opportunities for conducting long-term follow-up of participants and nonparticipants, overcoming challenges of low response rates typically associated with attrition from survey-based analyses of nonparticipants. The present study was done in connection with a follow-up study assessing mental and substance use disorders conducted during HUNT4. To assess differences between participants and nonparticipants, we applied a combined approach. First, we examined predictors of attrition in the follow-up study using information on sociodemographic and mental health characteristics from the main study. Second, we took advantage of the opportunity to link participation status in the follow-up study with complete data from patient registries to investigate health service use for mental health problems, including diagnostic information.

## Aim

The aim of the study was to increase knowledge about the representativeness of participants in a population-based diagnostic survey of mental disorders. We compared participants and nonparticipants with respect to (i) age, sex, education and income, (ii) general health, mental distress and risky alcohol use, (iii) use of primary and specialist health services for mental health problems, and (iv) prevalence of diagnosed mental disorders and substance use disorders recorded in the health services. We also examined associations of these predictors with nonparticipation status through regression analyses, controlling for sociodemographic characteristics.

## Materials and methods

### Study population and setting

#### The HUNT study

The study sample was based on a sub-sample of participants from the fourth survey of the Trøndelag Health Study (HUNT4), a population-based health survey conducted in 2017–2019 on the adult population (aged 20 years or older) in the county of Trøndelag in Mid-Norway. The previous three HUNT surveys were only conducted in the county of Nord-Trøndelag, but due to the unification Nord- and Sør-Trøndelag into one county (Trøndelag) in 2019, inhabitants in Sør-Trøndelag were also invited. Eligible participants received an invitation by mail or by SMS, with information about the survey and a code for accessing an online questionnaire containing questions about health, health complaints, disease and lifestyle. The participation rate was higher in Nord-Trøndelag (54%, *n* = 56 000) than in Sør-Trøndelag (42.6%, *n* = 105 900) [7]. All participants gave written consent to be contacted for follow-up studies in HUNT, and to have their survey information linked with data from former HUNT rounds, as well as administrative and health registries. The cohort profile, including sampling design and procedures, of the HUNT study is extensively described elsewhere [7].

#### Sampling and data-collection procedures for the diagnostic interview survey

A randomly selected sub-sample of HUNT4 participants aged 20 to 65 years from Nord-Trøndelag and the city of Trondheim (in Sør-Trøndelag) were invited to participate in a follow-up study assessing common mental and substance use disorders. Sampling was done from complete lists of all eligible participants who had given their written consent to be contacted for further studies. Younger persons were oversampled to compensate for expected nonparticipation in this part of the population. In Trondheim, males were also oversampled to constitute 58% of the first drawn sample, however, no gender-specific sampling was done in later draws.

Study information was posted to participants. In Nord-Trøndelag, they were asked to sign up for the survey by sending an email, SMS or letter to the local project coordinator. In Trondheim, they received their invitation to participate by SMS one week after they had received the information letter with a link containing information about the survey, and instructions to sign up for interviews by SMS or email. One reminder was sent by SMS to those who had not signed up approximately one week after the original invitation. All participants received a gift card of 300 NOK (around 30 dollars) for participation. Sampling and recruitment of participants continued until a total of 4000 interviews were completed, 2000 in Nord-Trøndelag and 2000 in Trondheim.

Mental and substance use disorders were assessed through face-to-face interviews using the 5th revision of the standardized Composite International Diagnostic Interview [18] (CIDI 5.0), which is also used in the World Mental Health Surveys [19]. The interviews were conducted at five local field stations spread around the county of Nord-Trøndelag from November 2018 to September 2019, and one field station in the city of Trondheim in Sør-Trøndelag from January to July 2020. Due to the outbreak of the COVID-19 pandemic, which hit Norway in late February 2020 and were met with social distancing measures from March 12th, 2020, 64% of the interviews in Trondheim were conducted over telephone. The participation rate in Trondheim was relatively similar before (29.8%) and during the first six months of the pandemic (range 28.7% to 32.8% between different periods) [20].

The present analyses are based on all invited persons to the follow-up survey. Information collected in HUNT4, as well as data from the Norwegian Population Registry and patient registries in the primary and specialist mental health services (covering the entire adult Norwegian population), were linked to the invited persons by their individual identification numbers and used to compare characteristics between participants and nonparticipants.

## Variables

### Sociodemographic characteristics

Data on recorded sex (binary male/female) and birth year was retrieved from the Norwegian Population Registry, while self-reported categorical information about marital status, highest attained education and total household income before tax was gathered in the HUNT4 survey. These categories are listed in Table 1. Being married, attaining four years of education or more at a University or University College, and total household income of more than 1 million Norwegian Kroner (NOK) were used as reference categories in their respective variables. Age at invitation to the survey was retrieved by subtracting survey invitation year from birth year, and categorized as 19 to 30 years, 31 to 45 years, and 46 to 68 years.

### General health, mental distress and risky alcohol consumption

General self-reported health was measured through the question “How is your health at the moment?”, with four item response categories, see Table 1. Self-reported mental distress in HUNT4 was measured using the CONOR Mental Health Index (CONOR-MHI). CONOR-MHI is a modified questionnaire based on the General Health Questionnaire (GHQ) [21] and the Hopkins Symptom Check List (HSCL-10) [22], as well as other mental distress screening questionnaires, and particularly developed for the Cohort of Norway Studies (CONOR). The instrument has been found to have high internal consistency (Cronbach’s alpha > 0.8), and strong correlations with the Hospital Anxiety and Depression Scale (HADS) and HSCL-10 [23]. It is a seven-item questionnaire with response categories on a 4-point Likert rating scale, with scores ranging from 1 to 4. Two items relating to calmness and optimism are reverse-coded when calculating the total score of the instruments. For respondents with missing responses on 1 or 2 items, the missing responses were imputed based on the mean score on the remaining valid items. Respondents with 3 or more missing items were set as “missing” for the whole scale. For the present study, we included the mean score with standard deviation (SD), as well as dichotomized variables based on the recommended cut off > = 2.15 [23]. The mean score across items is calculated by summing the score of each item and divide by seven, with higher score indicating more mental distress. The total score can range from 7 to 28, and mean score can hence range from 1 to 4.

Risky alcohol use was operationalized as binary variables, and assessed based on alcohol intake frequency (“about how often during the last 12 months did you drink alcohol”), where 2–3 times a week or more was used as cut-off, and frequency of binge drinking (“how often do you drink 6 glasses or more of beer, wine and spirits in one sitting”) with monthly or more as cut-off. Total alcohol consumption was calculated by summing the usual number of drinks over a fortnight period.

### Health service utilization and diagnoses

Information about contact with health services due to mental health problems was retrieved from the Norwegian Registry for Primary Health Care (NRPHC), and from the Norwegian Patient Registry (NPR), which contains information about use of specialist mental health care [24]. The NPR has information about all use of public specialist health care for adults aged 18 years and older, including outpatient and inpatient care, contract specialists and interdisciplinary addiction treatment, with diagnostic information coded according to the International Classification of Diseases 10th Revision (ICD-10) [25]. From NRPHC, we included information about contact with general practitioners and emergency rooms, with diagnoses primarily coded according to International Classification of Primary Care (ICPC-2) [26], with some categories coded according to ICD-10. For the present study, we included information about date of contact and for which diagnosis (primary and secondary diagnoses) the contact was attributed to. The registry data from NPR and NRPHC included in the present study only contained information about health service contact from age 18 and older. Hence, for some of the individuals in the younger tire of the study sample, information about health service contact more than 24 months before participation was not retrieved.

### Primary health care

We included all NRPHC recorded direct patient consultations at the physician premises, including general consultations, consultations regarding sick leave certification, individual psychotherapy and group therapy, and home visits. Records of indirect patient work, writing prescriptions, vaccination, biometrical tests, ultrasound and other procedures were excluded, as these were considered not being directly related to consultations for mental health problems. The majority of contacts were with general practitioners (97.5%), and 2.5% of contacts were with emergency rooms. The recorded diagnoses according to the F-chapter in ICD-10 or the P-chapter in the ICPC-2 were used as indication of the specific mental health problems discussed in the consultation and categorized according to the commonly used categories in diagnostic manuals and research practice. We included all diagnoses indicating a mental health problem recorded for each consultation. Hence, one individual could be recorded as a case in multiple diagnostic categories during one consultation. A person could have up to the maximum observed 400 consultations during the follow-up period (January 1 st 2018 to December 31 st 2021), ranging from 1,071 days before to 1,024 days after the invitation date. For the purposes of the present study, we chose to focus on the first consultation in three defined time periods (0–31 days, 31–365 days and 365 days or more before or after the invitation date) and extract the diagnostic codes from these consultations. These codes were used to inform whether the person was a case in each of the included diagnostic categories during the whole study period. Comorbidities were present if a person was identified in 2 or more diagnostic categories during the study period.

### Specialist mental health care

Contact with specialist mental health care recorded in NPR included all direct outpatient or inpatient contacts, excluding indirect patient work, training and contacts where type of contact was not specified. Individuals could have up to the maximum observed 3,041 records during the follow-up period ranging from January 2nd 2008 to Augst 31 st 2022, equivalent to the range of 4,536 days before to 1,440 days after invitation date. In line with the procedure for consultations in primary health care, we extracted the diagnostic information from the first contact in each of the defined three time-periods before or after invitation date. We included all diagnoses recorded for these consultations, both primary and secondary diagnoses, and categorized these according to sub-chapters in the F-chapter in ICD-10. Some specific diagnoses of particular interest were given individual categories, i.e. F30-F31 (manic episode and bipolar disorders), F32-F33 (depressive disorders), F50 (eating disorders) and F60-F61 (personality disorders). Diagnoses were coded in dichotomized categories that were not mutually exclusive, allowing individuals to be categorized under multiple diagnoses. Comorbidities were defined as being recorded with 2 or more diagnostic categories during the study period.

### Statistical analyses

Differences in characteristics between participants and nonparticipants were explored using descriptive statistics (numbers and percentages and means or medians with standard deviations (SD) or interquartile range (IQR) for continuous variables), and logistic regression analyses to assess associations between predictor variables and participation status as the outcome. Associations are presented as odds ratios (OR) with 95% confidence intervals (CI), both unadjusted and adjusted for sociodemographic variables (sex, age categories, marital status, income and education). All analyses were conducted using Stata SE 17.1 [27].

### Ethics

The HUNT4 survey was approved by the Regional Committee for Medical and Health Research Ethics (2017/28/REK Midt), as well as the data-collection for the interview survey. All HUNT4 participants provided written informed consent for their data to be linked with registry information.

## Results

### Sociodemographic predictors

Among the 16,599 HUNT4 participants invited, 4,220 participated in the follow-up psychiatric diagnostic interview survey, giving a participation rate of 25.4%. Sociodemographic characteristics and their associations with nonparticipation in the psychiatric diagnostic interview survey are presented in Table 1. Compared to their respective reference categories, males (OR for nonparticipation 1.29 (95% CI 1.20–1.39)), younger individuals (19–30 years: OR 1.30 (1.19–1.42) and 31–45 years: OR 1.29 (1.19–1.41)), being unmarried (OR 1.20 (1.11–1.29)) and having lower education and lower income were all identified predictors of nonparticipation. The strongest point estimates were found for lower education levels, e.g. primary education (OR 3.10 (2.41–3.98)). Being widowed, separated or divorced was not associated with participation status. A higher percentage among the nonparticipants compared to participants had missing information on marital status, educational attainment and income.

**Table 1:** Sociodemographic variables and mental health characteristics in HUNT by participation status in the psychiatric interview survey. The HUNT study (2017-19), Norway

	Participants *N* = 4,220	Nonparticipants *N* = 12,379	OR for nonparticipation
	*n* (%)	*n* (%)	OR (95% CI)
**Sex**			
Female	2,552 (60.5)	6,713 (54.2)	Ref.
Male	1,668 (39.5)	5,666 (45.8)	**1.29 (1.20–1.39)**
**Age/age-groups**			
Mean age (SD)	40.3 (12.8)	38.7 (12.1)	**0.99 (0.99–0.99)**
19–30 years	1,244 (29.5)	3,974 (32.1)	**1.30 (1.19–1.42)**
31–45 years	1,487 (35.2)	4,736 (38.3)	**1.29 (1.19–1.41)**
46–68 years	1,489 (35.3)	3,669 (29.6)	Ref.
**Marital status**			
Married	1,605 (38.0)	4,270 (34.5)	Ref.
Unmarried	2,257 (53.5)	7199 (58.2)	**1.20 (1.11–1.29)**
Widowed	21 (0.5)	60 (0.5)	1.07 (0.65–1.77)
Divorced, separated	329 (7.8)	767 (6.2)	0.88 (0.76–1.01)
Missing	8 (0.2)	83 (0.7)	-
**Highest attained education**			
Primary education	75 (1.8)	437 (3.5)	**3.10 (2.41–3.98)**
Upper secondary 1–2 years	225 (5.3)	1,001 (8.1)	**2.36 (2.02–2.77)**
Upper secondary 3 years	583 (13.8)	2,025 (16.4)	**1.85 (1.65–2.06)**
Vocational training	536 (12.7)	2,349 (19.0)	**2.33 (2.08–2.60)**
Higher education, < 4 years	1,128 (26.7)	2,899 (23.4)	**1.37 (1.25–1.50)**
Higher education, > 4 years	1,578 (37.4)	2,970 (24.0)	Ref.
Missing	95 (2.3)	698 (5.6)	-
**Income**			
Below 250,000 NOK	401 (9.5)	1,217 (9.8)	**1.33 (1.16–1.51)**
250,000–450,000 NOK	516 (12.2)	1,709 (13.8)	**1.45 (1.29–1.63)**
451,000–750,000 NOK	985 (23.3)	2,973 (24.0)	**1.32 (1.20–1.46)**
751,000–1,000,000 NOK	916 (21.7)	2,684 (21.7)	**1.28 (1.16–1.42)**
More than 1,000,000 NOK	1,272 (30.1)	2,909 (23.5)	Ref.
Missing	130 (3.1)	887 (7.2)	-
**Self-reported health**			
Poor	61 (1.5)	198 (1.6)	1.32 (0.99–1.78)
Not so good	652 (15.5)	1,915 (15.5)	**1.20 (1.07–1.34)**
Good	2,217 (52.5)	7,026 (56.8)	**1.29 (1.19–1.40)**
Very good	1,256 (29.8)	3,081 (24.9)	Ref.
*Missing*	34 (0.8)	159 (1.3)	
**Symptoms of mental distress (CONOR-MHI)**			
Score (mean, SD)	1.61 (0.54)	1.59 (0.52)	**0.91 (0.85–0.97)**
Above cut-off 2.15 (yes)	530(12.6)	1,444 (11.7)	0.94 (0.85–1.05)
*Missing*	39 (0.9)	367 (3.0)	n.a.
**Risky alcohol use**			
Alcohol consumption 2 times a week or more	988 (23.4)	2,261 (18.3)	**0.76 (0.70–0.83)**
*Missing*	66 (1.6)	573 (4.6)	n.a.
Binge drinking monthly or more	779 (18.5)	2,266 (18.3)	1.04 (0.95–1.14)
*Missing*	117 (2.8)	795 (6.4)	n.a.
Number of drinks per fortnight (mean, SD)	5.8 (7.0)	5.6 (6.8)	1.00 (0.99–1.00)
*Missing*	171 (4.1)	1,163 (9.4)	n.a.

### Health information from the main survey

Self-reported good or not-so-good health, but not poor health, was associated with nonparticipation compared to very good health. A higher score on CONOR-MHI was associated with lower odds of nonparticipation (OR 0.91 (0.85–0.97)), while dichotomizing on the cut-off was not associated with participation status. In regard to reported risky alcohol consumption in HUNT4, alcohol consumption two times a week or more was associated with lower odds of nonparticipation (OR 0.76 (0.70–0.83)), while number of units per fortnight and binge drinking monthly or more often was not associated with participation (Table 1).

### Contact with primary and specialist health care for mental health problems

Table 2 presents the associations between participation status in the follow-up survey and contact with primary and specialist mental health care for mental health problems. Among the participants, 51.4% had consultations in primary health care for mental health problems, 20.8% had outpatient contact and 3.4% had inpatient contact in the specialist mental health care. Corresponding numbers among nonparticipants were 48.2% (primary health care), 16.9% (outpatient) and 3.0% (inpatient). Regardless of the time frame from invitation date, individuals with primary health care consultations or outpatient contacts in specialist mental health services had lower odds for nonparticipation than individuals who were not in contact with primary or specialist mental health services. The crude OR for contact with primary health care zero to 1,071 days before or after invitation date was 0.88 (0.82–0.94), and for outpatient contact zero to 4,536 days before or after invitation date was 0.78 (0.71–0.85). The associations were consistent across different timeframes (0 to 31 days, 32–365 days, and more than 365 days) with overlapping CIs, and were strengthened when adjusted for sociodemographic variables (adjusted OR 0.83 (0.77–0.89) and 0.68 (0.62–0.75) for primary health care and outpatient contact in specialist mental health care, respectively). Inpatient contact with specialist mental health care was not associated with participation status in the crude analyses. When adjusted for sociodemographic variables, inpatient contact more than 365 days before or after invitation date to the survey, was associated with lower odds for nonparticipation (OR 0.65 (0.53–0.80)). There was no association between number of days hospitalized and participation status.

**Table 2:** Participation status and contact with primary and specialist healthcare for mental and substance use disorders in three time periods from invitation date (before or after).*

Days from invitation date	Participants *N* = 4,220	Nonparticipants *N* = 12,379	Crude OR for nonparticipation	Adjusted OR for nonparticipation^3^
	n (%)	n (%)	OR (95% CI)	OR (95% CI)
Primary health care				
0–31 days	366 (8.7)	884 (7.1)	**0.81 (0.71–0.92)**	**0.76 (0.67–0.87)**
32–365 days	1,373 (32.5)	3,748 (30.3)	**0.90 (0.84–0.97)**	**0.85 (0.78–0.92)**
More than 365 days^1^	1,729 (41.0)	4,593 (37.1)	**0.85 (0.79–0.91)**	**0.81 (0.75–0.87)**
Total	2,170 (51.4)	5,964 (48.2)	**0.88 (0.82–0.94)**	**0.83 (0.77–0.89)**
Specialist health care – outpatient contact				
0–31 days	143 (3.4)	293 (2.4)	**0.69 (0.56–0.85)**	**0.60 (0.48–0.74)**
32–365 days	347 (8.2)	765 (6.2)	**0.74 (0.64–0.84)**	**0.63 (0.55–0.72)**
More than 365 days^2^	827 (19.6)	1,965 (15.9)	**0.77 (0.71–0.85)**	**0.68 (0.62–0.75)**
Total	876 (20.8)	2,090 (16.9)	**0.78 (0.71–0.85)**	**0.68 (0.62–0.75)**
Specialist health care – inpatient contact				
0–31 days	5 (0.1)	24 (0.2)	1.64 (0.62–4.29)	1.28 (0.48–3.44)
32–365 days	28 (0.7)	97 (0.8)	1.18 (0.78–1.80)	0.83 (0.53–1.28)
More than 365 days^2^	137 (3.3)	338 (2.7)	0.84 (0.68–1.02)	**0.63 (0.51–0.78)**
Total	144 (3.4)	367 (3.0)	0.86 (0.71–1.05)	**0.65 (0.53–0.80)**
Number of days hospitalized (median, IQR)^4^	26.5 (71)	33 (104)	1.00 (1.00–1.00)	1.00 (1.00–1.00)

*Individuals may have had contacts in several time periods, hence the numbers for each period cannot be added up to a total. ^1^Up to 1,189 days from invitation date.^2^Up to 4,526 days from invitation date.^3^Adjusted for sex, age-category, marital status, income and education.^4^Among those hospitalized.

### Recorded diagnoses in primary and specialist health care

The prevalence of diagnoses from primary and specialist mental health services among participants and nonparticipants, and the crude and adjusted associations with non-participation are listed in Table 3.

**Table 3:** Mental disorders among participants and nonparticipants in the psychiatric interview study. Recorded diagnoses from primary health care and specialist health care during the total follow-up period

	Participants *n* = 4,220	Nonparticipants *N* = 12,379	OR for nonparticipation	
	*n* (%)	*n* (%)	Crude OR (95% CI)	Adjusted OR (95% CI)
Primary health care^1^				
Organic mental disorders (F00-F09)	85 (2.0)	241 (2.0)	0.97 (0.75–1.24)	0.93 (0.72–1.21)
Substance use disorders (F10 – F19)	84 (2.0)	222 (1.8)	0.90 (0.70–1.16)	0.89 (0.68–1.15)
Schizophrenia and psychotic disorders (F20-F29)	160 (3.8)	464 (3.8)	0.99 (0.82–1.19)	0.99 (0.82–1.20)
Intellectual idiopathic development disorders (F70-F79)	412 (9.8)	1,131 (9.1)	0.93 (0.83–1.05)	0.90 (0.80–1.02)
Developmental disorders, incl. ASPD (F80-F89)	10 (0.2)	33 (0.3)	1.13 (0.55–2.29)	1.11 (0.54–2.28)
Milder mental health complaints (ICPC P01-P29)	1,197 (28.4)	3,286 (26.5)	**0.91 (0.84–0.99)**	**0.90 (0.83–0.98)**
ICPC P73: Affective disorder	45 (1.1)	54 (0.4)	**0.41 (0.27–0.60)**	**0.31 (0.20–0.48)**
ICPC P76: Depressive disorder	330 (7.8)	821 (6.6)	**0.84 (0.73–0.96)**	**0.82 (0.71–0.94)**
ICPC P74, P79, P82: Anxiety disorders	164 (3.9)	504 (4.1)	1.05 (0.88–1.26)	0.96 (0.80–1.17)
ICPC P80: Personality disorder	26 (0.6)	56 (0.5)	0.73 (0.46–1.17)	**0.52 (0.32–0.85)**
ICPC P81: Hyperkinetic disorder	82 (1.9)	172 (1.4)	**0.71 (0.55–0.93)**	**0.54 (0.41–0.72)**
Other ICPC diagnoses from the P-Chap. 2	66 (1.6)	210 (1.7)	1.09 (0.82–1.44)	0.89 (0.67–1.20)
Comorbidities				
1 diagnosis	1,683 (39.9)	4,705 (38.0)	Ref.	Ref.
2 or more diagnoses	485 (11.5)	1,252 (10.1)	0.92 (0.82–1.04)	0.88 (0.78–1.00)
Specialist mental health care^3^				
Substance use disorders (F10-F19)	35 (0.8)	174 (1.4)	**1.70 (1.18–2.46)**	1.16 (0.79–1.69)
Schizophrenia and psychotic disorders (F20-F29)	10 (0.2)	46 (0.4)	1.57 (0.79–3.11)	1.24 (0.59–2.60)
Manic episode and bipolar disorders (F30-F31)	49 (1.2)	79 (0.6)	**0.55 (0.38–0.78)**	**0.49 (0.33–0.72)**
Depressive disorders (F32-F33)	305 (7.2)	680 (5.5)	**0.75 (0.65–0.86)**	**0.73 (0.63–0.85)**
Neurotic and anxiety disorders (F40-F48)	452 (10.7)	1,061 (8.6)	**0.78 (0.70–0.88)**	**0.72 (0.63–0.81)**
Eating disorders (F50)	34 (0.8)	81 (0.7)	0.81 (0.54–1.21)	0.85 (0.56–1.30)
Personality disorders (F60-F61)	92 (2.2)	209 (1.7)	**0.77 (0.60–0.99)**	**0.92 (0.48–0.80)**
Developmental disorders, incl. ASPD (F80-F89)	34 (0.8)	95 (0.8)	0.95 (0.64–1.41)	0.68 (0.45–1.03)
ADHD and conduct disorder (F90-F91)	107 (2.5)	229 (1.9)	**0.72 (0.57–0.91)**	**0.56 (0.43–0.71)**
Other mental disorders^4^	72 (1.7)	198 (1.6)	0.94 (0.71–1.23)	0.84 (0.63–1.13)
Comorbidities				
1 diagnosis	645 (15.3)	1,560 (12.6)	Ref.	Ref.
2 or more diagnoses	245 (5.8)	570 (4.6)	0.97 (0.81–1.15)	0.87 (0.72–1.05)

^1^Follow-up period from January 1^st^ 2018 to December 31^st^ 2021. ^2^ Other ICPC diagnoses include P62, P70, P71, P72, P75, P77, P78, P85, P86, P98, P99. ^3^Follow-up period January 2^nd^ 2008 to Augst 31^st^ 2022. ^4^“Other mental disorders” include F00-F09, F34, F38, F39, F51-F59, F62-F69, F70-F79, and F92-F99

Among both participants and nonparticipants, the highest prevalences were found for primary health care diagnoses of milder mental health complaints (28.4% and 26.5% among participants and nonparticipants, respectively), intellectual idiopathic development disorders (9.8% and 9.1%) and depressive disorder (7.8% and 6.6%). Having an ICPC diagnosis for milder mental health complaints (OR 0.91 (0.84–0.99), affective disorder (OR 0.41 (0.27–0.60)), depressive disorder (OR 0.84 (0.73–096)) or hyperkinetic disorder (OR 0.71 (0.55–0.93)) was associated with lower odds for nonparticipation, with associations further strengthened when adjusting for sociodemographic variables, for which a significant association was also found for personality disorder (OR 0.52 (0.32–0.85)) (Table 3). ICD-10 diagnoses in primary health care for organic mental disorders, substance use disorders, schizophrenia and psychotic disorders, intellectual idiopathic developmental disorders, and developmental disorders, were not associated with participation status, neither was an ICPC diagnosis indicating anxiety disorders or the “other” category of ICPC diagnoses. Compared with only one recorded diagnosis, comorbidity was not associated with participation status (Table 3).

In specialist mental health care (outpatient and inpatient combined), the most prevalent diagnoses among participants and nonparticipants respectively were neurotic and anxiety disorders (10.7% and 8.6%) and depressive disorders (7.2% and 5.5%) (Table 3). Having an ICD-10 diagnosis of manic episode and bipolar disorders (OR 0.55 (0.38–0.78)), depressive disorders (OR 0.75 (0.65–0.86)), neurotic and anxiety disorders (OR 0.78 (0.70–0.88)), personality disorders (0.77 (0.60–0.99)) or ADHD and conduct disorder (OR 0.72 (0.57–0.91)) was associated with lower odds of nonparticipation, with associations strengthened when adjusting for sociodemographic variables. In contrast, substance use disorders were associated with higher odds for nonparticipation (OR 1.70 (1.18–2.46)), but the association disappeared when adjusting for sociodemographic variables. Schizophrenia and psychotic disorders, eating disorders, developmental disorders and the “other mental disorders” category showed no association with participation, neither did comorbidities.

## Discussion

In this study, based on a sub-sample of HUNT4 participants, we examined the characteristics of participants and nonparticipants in a follow-up psychiatric diagnostic survey using self-reported information from the main survey and linked registry data. Being male, younger, unmarried, or having lower educational attainment and income were associated with higher odds for nonparticipation. Contact with the primary or specialist outpatient health services, or inpatient services more than a year from invitation date, was associated with lower odds for nonparticipation. Diagnoses indicating affective disorder, anxiety disorder, personality disorder, hyperkinetic disorder or other milder mental health complaints were also associated with lower odds for nonparticipation.

These results stand in contrast to several other studies finding a higher risk of nonparticipation in population-based health surveys among individuals with mental health complaints [7, 11–14, 16, 17]. For instance, in the recent Danish study linking invitation status and health registry records, Rygner et al. found strong associations between psychiatric hospitals diagnoses and nonparticipation, with nonparticipants having more than twice the prevalence of any psychiatric diagnosis compared to participants [17]. Underrepresentation was even higher among individuals with severe mental disorders with three times higher prevalence of substance use disorders and psychotic disorders, and 11 times higher prevalence of organic disorders/dementia among nonparticipants compared with participants. Additionally, nonparticipants had 40% to 75% higher prevalence of more common mental disorders, such as neurotic disorders and depressive episode than participants. In contrast, we found no association between participation status for organic disorders, substance use disorders or psychotic disorders. In addition, participants in our follow-up study had a 20% to 30% higher prevalence of recorded diagnoses of common mental disorders in both primary and specialist health care compared to nonparticipants. Interestingly, in contrast to the findings by Rygner et al., the association between prior contact with mental health services and participation in our psychiatric interview survey became even more pronounced after adjusting for sociodemographic factors. This pattern indicates that these factors may have obscured the strength of the association in the unadjusted model.

It is likely that health-related self-selection primarily happened during the initial recruitment to HUNT4, in which the entire population of Trøndelag county above age 20 was approached, and not from the subsample of HUNT participants invited to the subsequent psychiatric interview study. Nonparticipation analyses from the initial HUNT4 showed lower probability of participation among persons not married or registered partners, having BMI over 30, having poorer self-reported health, being current smokers, having a history of chronic diseases, or having sought health care for mental health problems [7]. Using registry data to compare the HUNT participants with the general population of the Trøndelag county, there were only minor differences in number of visits to general practitioners. However, depending on age and sex, the prevalence of anxiety and depression were between 0% and 20% higher, and diagnosed alcohol abuse twice as high, in the general population compared to HUNT participations [7]. In summary, this may indicate that the sub-sample invited to the psychiatric interview survey was healthier than the general population. Further, recruitment from the pool of participants in HUNT may also give a bias towards individuals with greater interest and willingness to participate in surveys than the general population. These individuals may also be more aware or invested in their mental health, or more likely to follow through other things, such as contacting the health care system when they experience health problems. With a participation rate in the interview study of 25.4%, other factors are also likely to influence the decision to participate, such as survey fatigue [28] or whether the topic of the survey felt relevant or interesting [29]. People with lived experiences of mental health problems, may be more motivated to contribute to topic specific research. Studies have also found higher participation rates in face-to-face interviews compared to self-administered questionnaires [30]. It has been argued that people who participate in studies focused on mental health may seek a therapeutic environment, in particular if the data is collected through face-to-face interviews [31]. Even tough we emphasized the scientific aim of assessing the mental health of the general population during the recruitment procedures, we cannot exclude the possibility that participants could also be motivated by the chance to talk about their mental health. Taken together, the finding that participants in HUNT4 appear to have better overall health than nonparticipants, suggests an initial selection bias based on health status. Within this context, individuals with poor health who do participate may represent a selected subgroup, namely those with sufficient capacity to engage in population-based studies despite health challenges. Thus, while the initial selection in HUNT4 likely reflects capacity to participate, subsequent participation in follow-up studies may be more influenced by interest in the study topic. Specifically for mental health outcomes, HUNT4-participants with mental health challenges may represent a subgroup with sufficient capacity to engage in population-based research. Among these, those who chose to participate in the current sub-study may have done so due to heightened interest or personal connection to the research topic. This step-wise selection mechanism may help reconcile our findings with earlier literature [7, 11–14, 16, 17].

The findings from the present study might be worth considering alongside signals of increasing prevalence of mental health complaints and depression and anxiety disorders found in mental health surveys in high-income settings [32–34]. If over time ‘true cases’ of mental disorders are more likely to participate in mental health surveys than non-cases, this could be contributing to data indicating a rise in prevalence. The present study cannot answer this, but it should be a topic for future research. The findings also indicate that specific selection bias in population-based surveys may differ over time and between contexts. Hence, it is important to do individual assessments of predictors of nonparticipation in each survey and develop survey specific weights or adjustment methods to compensate for these biases [15, 35].

## Strengths and limitations

The main strength of this study was the possibility to link survey information and health care records on an individual level among all invited persons to the psychiatric diagnostic survey, with a long follow-up time. We had access to complete data on health service use and diagnostic records spanning up to twelve years before and four years after the invitation date. Additionally, the inclusion of all recorded diagnoses from consultations over the follow-up period allowed us to assess association with nonparticipation across a broad range of diagnostic categories, and comorbidities. Access to diagnostic records from primary health care is also a major strength of this study.

However, the study also has important limitations. Most importantly, we could only compare characteristics within our subsample of HUNT4 participants, and not among the entire population originally invited to HUNT4 within the relevant age-range. The sampling procedure, which was introduced to ensure enough power among groups with known tendencies of higher nonparticipation (e.g. males and younger age groups), precludes comparisons of sociodemographic characteristics with the general population, as this will also affect the distribution of marital status, educational attainment and income. Further, the recruitment strategies differed somewhat between Nord- and Sør-Trøndelag (Trondheim), which may have influenced participation rates in these regions. Also, the data-collection in Trondheim coincided with the outbreak of the COVID-19 pandemic and the introduction of social distancing measures. This may have impacted both overall participation rates and participation rates among individuals with mental health problems specifically. However, as presented in a previous publication, we found no significant differences in neither participation rates nor prevalence of mental health problems among participants before and during the first six months of the pandemic [20]. Finally, as we only had access to registry data on people aged 18 years and older, we could not examine differences in mental health problems in childhood and adolescence among participants and nonparticipants. Further, knowledge about diagnostic history and health service use before age 18 was not captured in the present study, which might give an age-bias to the analyses based on the more than 12 months time-span for the youngest tire of our sample. However, examining the rates of health service use more than 12 months before invitation date between individuals aged 19 to 25, and those 26 and older, we found no difference in hospitalization and use of primary healthcare (*p* > 0.05), and somewhat higher proportion of outpatient specialist heath care use among those aged 19 to 25 (*p* = 0.001). Hence, we found no evidence of an underrepresentation of the younger tire for these analyses.

## Conclusion

The results from the current study indicated that having been in contact with the health services for a range of common mental health complaints or disorders, increased the chance of participating in a survey focused on assessing mental disorders. The results deviate from previous studies finding higher rates of nonparticipation among those with mental health problems and demonstrate that the direction of selection bias should be carefully considered in population-based studies. Even if people with mental health problems are often under-represented in surveys, this may not always be the case and needs to be carefully considered for adjustment and interpretation in each survey.

## Data Availability

Norwegian data protection regulations and GDPR impose restrictions on sharing of individual participant data. However, researchers may gain access to survey participant data by applying for HUNT data (www.ntnu.edu/hunt/datat) and registry data (www.helsedata.no). Approval from the Norwegian Regional Committee for Medical and Health Research Ethics (https://helseforskning.etikkom.no) is a pre-requirement for access to the data. Analytic codes for the analyses are available upon request to the corresponding author.

## Abbreviations

CONOR-MHI: CONOR Mental Health Index
GHQ: General Health Questionnaire
HADS: Hospital Anxiety and Depression Scale
HSCL-10: Hopkins Symptom Check List
ICD-10: International Classification of Diseases 10th Revision
ICPC-2: International Classification of Primary Care
NEMESIS: Netherlands Mental Health survey and Incidence Study
NRPHC: Norwegian Registry for Primary Health Care
NPR: Norwegian Patient Registry
SHOT: Students` Health and Wellbeing Study
HUNT: Trøndelag Health Study
